# Supplementary material for: Electronic prescribing systems as tools to improve patient care: a learning health systems approach to increase guideline concordant prescribing for venous thromboembolism prevention
Source: BMC Med Inform Decis Mak. 2022 May 3;22:121. doi: 10.1186/s12911-022-01865-y (PMC9066759; doi:10.1186/s12911-022-01865-y)
Supplement: Supplementary file 1 — Additional file 1. Statistical methods for z-score calculations. [file 12911_2022_1865_MOESM1_ESM.docx]

**Electronic prescribing systems as tools to improve patient care: a learning health systems approach to increase guideline concordant prescribing for venous thromboembolism prevention.**

**Online supplement**

S. Gallier, A. Topham, P. Nightingale, M. Garrick, I. Woolhouse, M.A. Berry, T. Pankhurst,

E. Sapey, S. Ball

**Statistical methods for z score calculations.**

If doctor “*I”* correctly treats “r*_i_*” cases from “n*_i_”* cases the expected proportion p of correct responses across all doctors is $p=\frac{sum\left( r_{i} \right)}{sum\left( n_{i} \right)}$ and has standard deviation $\sqrt{\frac{p\left( 1-p \right)}{sum\left( n_{i} \right)}}$. The observed proportion of correctly treated patients by doctor *i* is $p_{i}=\frac{r_{i}}{n_{i}}$ and has standard deviation$\sqrt{\frac{p\left( 1-p \right)}{n_{i}}}$. The standard deviation uses the expected proportion p of correct responses across all doctors and number n*_i_* cases treated by doctor *i.* The z score is $z=\frac{\left( p_{i}- p \right)}{\sqrt{\left( p\left( 1-p \right)*\left( \frac{1}{n_{i}}+\frac{1}{sum\left( n_{i} \right)} \right) \right)}}$. When sum(n*_i_*) is much larger than any n*_i_* the z-score may be simplified to $z=\frac{p_{i}-p}{\sqrt{\frac{p\left( 1-p \right)}{n_{i}}}}$. If z-scores are independent, the sum of *k* z-scores has mean 0 and standard deviation$\sqrt{k}$.
